# Five new coexisting species of copepod crustaceans of the genus *Spaniomolgus* (Poecilostomatoida: Rhynchomolgidae), symbionts of the stony coral *Stylophorapistillata* (Scleractinia)

**DOI:** 10.3897/zookeys.791.28775

**Published:** 2018-10-22

**Authors:** Mercedes Conradi, Eugenia Bandera, Sofya V. Mudrova, Viatcheslav N. Ivanenko

**Affiliations:** 1 Laboratorio de Biología Marina, Departamento de Zoología, Facultad de Biología, Universidad de Sevilla, Reina Mercedes 6, 41012, Sevilla, Spain Universidad de Sevilla Sevilla Spain; 2 Red Sea Research Center, King Abdullah University of Science and Technology (KAUST), Thuwal, 23955, Saudi Arabia King Abdullah University of Science and Technology Thuwal Saudi Arabia; 3 Department of Invertebrate Zoology, Faculty of Biology, Lomonosov Moscow State University, Moscow, 119992, Russia Lomonosov Moscow State University Moscow Russia

**Keywords:** Copepoda, Crustacea, symbiosis, biodiversity, Pocilloporidae, coral reefs, Red Sea

## Abstract

*Spaniomolgus* is a symbiotic genus of copepods of the poecilostomatoid family Rhynchomolgidae and is known to be associated with shallow-water reef-building hermatypic corals. Three species of this genus were previously found only in washings of *Acropora* and *Stylophora* in northern Madagascar. Four coral morphotypes of *Stylophorapistillata* (Pocilloporidae) were collected by SCUBA at 1 to 28 m depth in five sites in the Saudi Arabian Red Sea in 2013. Copepods found on these colonies were studied using light, confocal and scanning electron microscopy. Five new, and one known, species of the genus *Spaniomolgus* were discovered in washings and inside the galls of the hermatypic coral *S.pistillata*. The description of these new species (*Spaniomolgusglobus***sp. n.**, *S.stylophorus***sp. n.**, *S.dentatus***sp. n.**, *S.maculatus***sp. n.**, and *S.acutus***sp. n.**) and a key for the identification of all of its congeners is provided herein.

## Introduction

Rhynchomolgidae Humes and Stock, 1973 is one of the largest families of poecilostomatoid copepods comprising over 250 species living in association with various marine invertebrates (Ho and Kim 2001; Boxshall and Halsey 2004). There are 44 genera in the family Rhynchomolgidae with the genus *Doridicola* Leydig, 1853 being the largest in the family and comprising 52 species (Ho and Ivanenko 2013, Walter and Boxshall 2018). Thirty-eight genera of the family include only up to six species. One of these small genera, *Spaniomolgus* Humes & Stock, 1973, consists of three species: the type species *S.compositus* (Humes & Frost, 1964), *S.geminus* (Humes & Ho, 1968) and *S.crassus* (Humes & Ho, 1968), all previously attributed to the genus *Lichomolgus* Thorell, 1859. *Spaniomolgus* are found in association with scleractinians of the genera *Acropora* Oken, 1815, *Seriatopora* Lamarck, 1816, and *Stylophora* Schweigger, 1820 from Madagascar (Humes and Ho 1968, Humes and Stock 1972, 1973). There have been no records of *Spaniomolgus* since the revision of the lichomolgoid complex (Humes and Stock 1972, 1973) and until the discovery of an unidentified species of *Spaniomolgus* living in modified polyps (galls) of *Stylophorapistillata* Esper, 1797 in the Red Sea (Ivanenko et al. 2014, Shelyakin et al. 2018).

Branching corals of *Stylophorapistillata* are widely distributed around the Indo-Pacific and are phenotypically plastic, i.e., morphological variation across different habitats, depths, and geographic regions can be observed. The latest study based on seven DNA loci demonstrated that *Stylophora* corals from the Red Sea belong to a single molecular clade, and that morphospecies of *Stylophorapistillata*, *S.danae* Milne Edwards & Haime, 1850, *S.subseriata* (Ehrenberg, 1834), and *S.kuehlmanni* Scheer & Pillai, 1983 from the Red Sea are now considered as synonyms of *S.pistillata* (Arrigoni et al. 2016).

This paper describes five new species of *Spaniomolgus* living in symbiosis with four morphotypes of *Stylophorapistillata* from the Red Sea. Comments on the relationships with other congeners are given, and a key to the species of the genus *Spaniomolgus* is presented.

## Materials and methods

The sampling was undertaken in accordance with the policies and procedures of the King Abdullah University of Science and Technology (KAUST). Permissions for KAUST to undertake the research were obtained from the appropriate governmental agencies of the Kingdom of Saudi Arabia.

**Figure 1.**
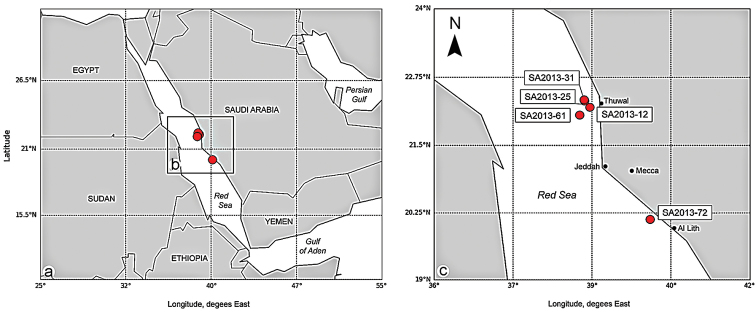
**a–c** Sampling localities and study area in the Red Sea (Saudi Arabia). The red circles indicate sampling localities of the indicated samples of *Stylophorapistillata* (see Table 1).

Four colonies of *Stylophorapistillata* from the Thuwal reefs in the central Red Sea and one colony from the reef close to Al Lith in the southern Red Sea were sampled (distance between the sampling locations is about 280 km) (Fig. 1, Table 1). The map was created using Python scripts (Jones et al. 2001), labels were included using the software Adobe Photoshop CS4 (Adobe Systems, San Jose, CA, USA). The coral colonies were collected using a hammer and chisel, and encased in sealed plastic bags while snorkeling and SCUBA diving at depths ranging from 1 to 28 m. The coral samples were rinsed on board as follows: 96% ethanol was added to each sample until the overall solution reached a concentration 10% to relax the animals attached to the coral. After 15 minutes, the samples were shaken, and the water with the detached symbionts was filtered through a 100 μm sieve. Copepods were sorted under a Carl Zeiss™ Stemi 2000-C stereomicroscope. Coral colonies were also examined for copepods in modified corallites and galls. Galls were dissected, and copepods were extracted from inhabited polyps using entomological needles and preserved in 96% ethanol.

**Table 1.** Sampling localities in the Red Sea.

**Specimen of the coral host**	**Species**	**Coordinates**	**Locality**	**Depth (m)**	**Date**
SA13-12	* Stylophora pistillata *	22°12'4.30"N, 38°57'31.40"E	Thuwal	1	24.04.2013
SA13-25	*Stylophorapistillata* (morphotype *subseriata)*	22°19'9.26"N, 38°51'15.78"E	Thuwal	10.4	25.04.2013
SA13-31	*Stylophorapistillata* (morphotype *danae)*	22°20'23.45"N, 38°50'52.33"E	Thuwal	28	26.04.2013
SA13-61	* Stylophora pistillata *	22°03'48.5"N, 38°45'51.2"E	Thuwal	1	29.04.2013
SA13-72	*Stylophorapistillata* (morphotype *mordax)*	20°08'02.1"N, 40°05'58.86"E	Al Lith	2.5	03.05.2013

**Figure 2.**
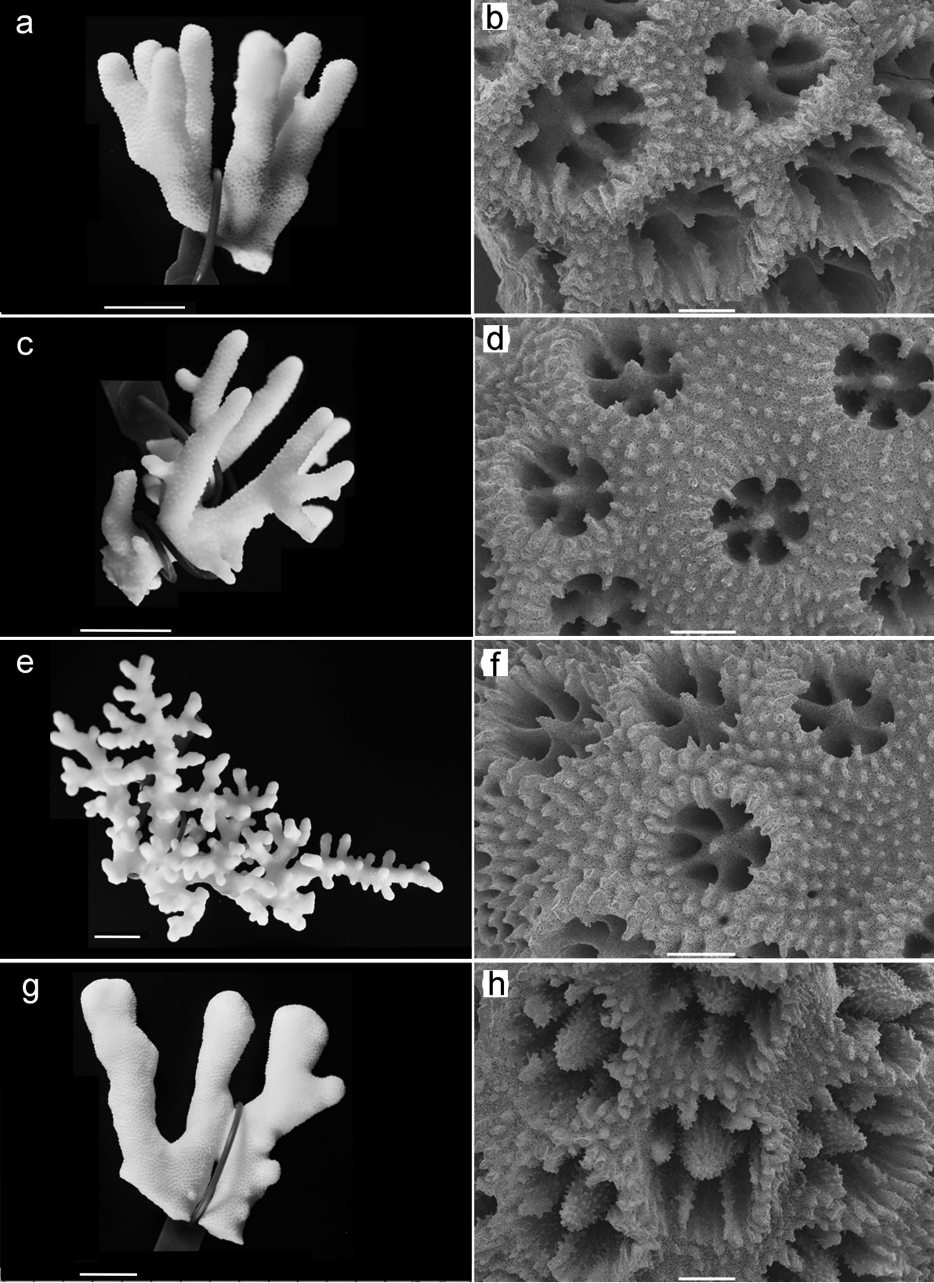
*Stylophorapistillata*, coral skeletons and corallite structures (SEM). **a, b** Specimen SA13-12 **c, d** Morphotype *subseriata*, specimen SA13-25 **e, f** Morphotype *danae* SA13-31 **g, h** Morphotype *mordax*, specimen SA13-61. Scale bars: 20 mm (**a, c, e, g**); 0.5 mm (**b, d, f, h**).

In the lab, copepods were dissected in lactic acid and then stained with Chlorazol black E (Sigma C-1144) for contrast enhancement (Ivanenko and Defaye 2004). Specimens were then examined as temporary mounts in lactophenol and later sealed with Entellan as permanent mounts. The coral hosts (Fig. 2) were bleached in sodium hypochlorite for 48 h, rinsed with fresh water, dried and photographed. The copepods were kept in 2 mL vials in 96% ethanol with a small drop of glycerol.

For confocal microscopy, exoskeletons were individually transferred to distilled water and then stained with Fuchsin (Ivanenko et al. 2012; Corgosinho et al. 2018). The copepods were inspected using an inverted Nikon A1 confocal laser scanning microscope (CLSM, Nikon Corporation, Tokyo, Japan) at Lomonosov Moscow State University, using a 40× oil immersion objective and lasers with wavelengths of 532 and 640 nm. The laser power was set to 60%. The amplitude offset and detector gain were manually adjusted. CLSM image stacks were obtained throughout the whole animal, and the scanning software was adjusted to perform the optimal number of scans. Image size was set for 2000×2000 dpi and the reconstruction of the external anatomy was obtained by maximum projection. The final images were adjusted for contrast and brightness using the software Adobe Photoshop CS4.

All figures were prepared using a Leica DM5500B differential interference microscope equipped with a camera lucida. The armature formula of swimming legs 1–4 follows Sewell (1949), spines are indicated by Roman numerals and setae by Arabic numerals. Mean body length (MBL) of copepods was measured from the anterior margin of the rostrum to the posterior margin of the caudal rami.

For scanning electron microscopy (SEM), copepods were dehydrated through increasing ethanol concentrations, critical point dried, mounted on aluminium stubs, coated with gold, and examined in a CamScan SEM (CamScan Electron Optics Ltd, London, UK) at the Faculty of Biology of Lomonosov Moscow State University. The bleached fragments of corals were mounted on metal stands using glue, coated with a conductive gold film and examined with the same SEM.

Type specimens of copepods are deposited in the collection of the Zoological Museum, Moscow Lomonosov State University (ZMMU). The coral hosts are deposited in the collection of King Abdullah University of Science and Technology (KAUST).

## Results

Five new and one described species of the genus *Spaniomolgus* were found in washings and inside of polyps of four morphotypes of the hermatypic coral *Stylophorapistillata* collected from five sites (Table 1, Fig. 1) at depths ranging from 1 to 28 m. The description of the five new species (*Spaniomolgusglobus* sp. n., *S.stylophorus* sp. n., *S.dentatus* sp. n., *S.maculatus* sp. n., and *S.acutus* sp. n.) is provided herein.

### Taxonomy

#### Poecilostomatoida Thorell, 1859

##### Family Rhynchomolgidae Humes & Stock, 1973

###### Genus Spaniomolgus

Taxon classificationAnimaliaCyclopoidaRhynchomolgidae

Humes & Stock, 1973

####### Type species.

*Lichomolguscompositus* Humes & Frost, 1964 now regarded as a synonym of *Spaniomolguscompositus* (Humes & Frost, 1964), by original designation.

####### Other species.

*Spaniomolgusgeminus* (Humes & Ho, 1968), *S.crassus* (Humes & Ho, 1968), *S.globus* sp. n., *S.stylophorus* sp. n., *S.dentatus* sp. n., *S.maculatus* sp. n., *S.acutus* sp. n.

####### Remarks.

The publication by Humes and Stock in 1972 of a list of new taxa, including *Spaniomolgus* and Rhynchomolgidae, without diagnoses of the new taxa is considering by us as interrupted and continued in 1973 (ICZN 1999: Art. 10.1.1); therefore the publication date of the genus becomes 1973.

###### Spaniomolgus globus sp. n.

Taxon classificationAnimaliaCyclopoidaRhynchomolgidae

http://zoobank.org/9EC98428-E87D-4854-B2C7-7BEAA59DF14A

Figs 3 , 4

####### Type locality.

Saudi Arabian Red Sea, reef near Thuwal, 22°03'48.5"N, 38°45'51.2"E.

####### Material examined.

1 ♀ holotype (ZMMU Me-1209) and 3 ♀♀ paratypes (ZMMU Me-1210) from tubular-shaped modification of corallites of *Stylophorapistillata* (KAUST SA2013-61) collected at 1 m depth.

####### Etymology.

The specific Latin epithet *globus*, globe, refers to the body shape in life when the urosome forms an s-shaped flexure.

####### Description.

Adult female.

**Figure 3.**
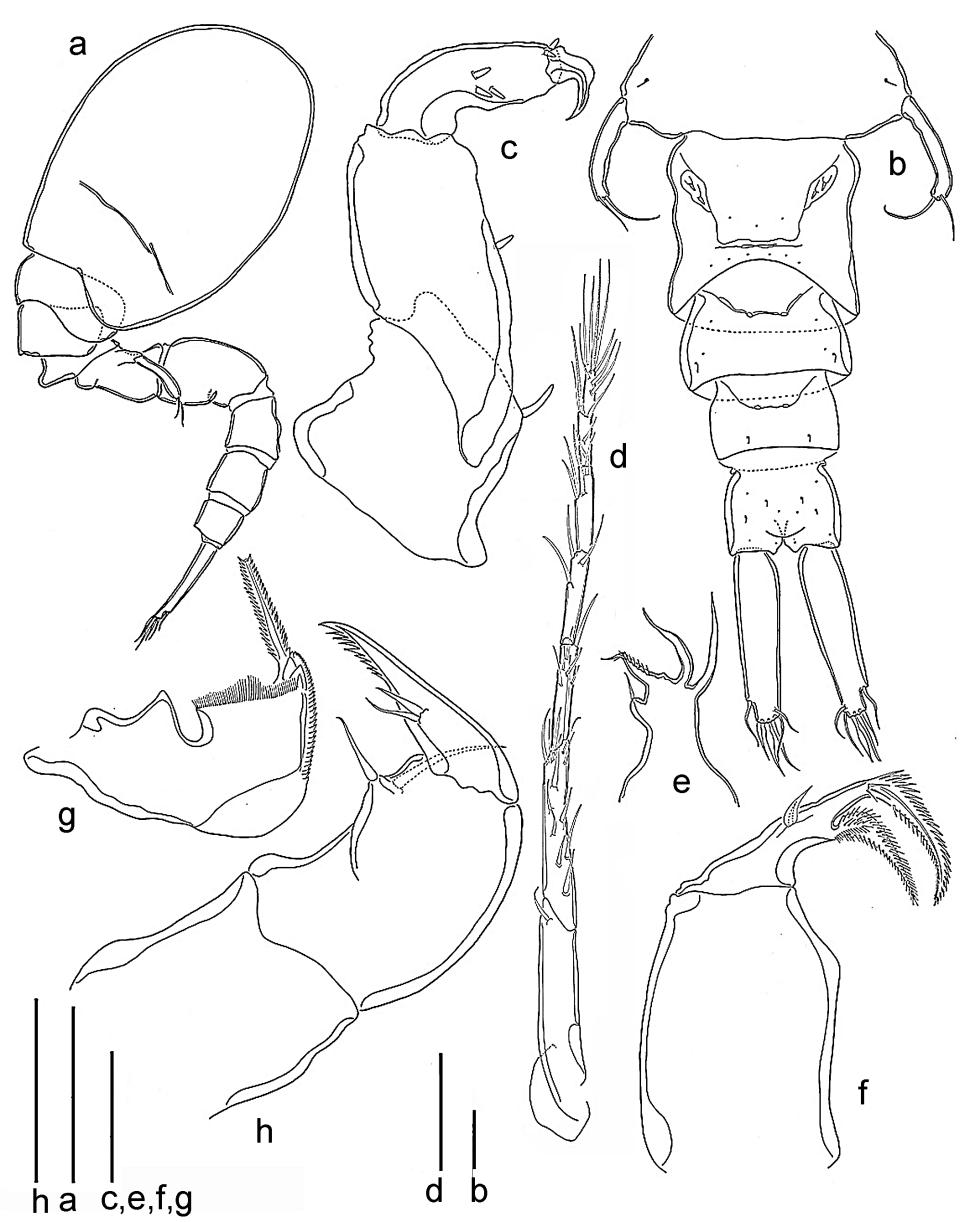
*Spaniomolgusglobus* sp. n., female. **a** Habitus lateral **b** Urosome dorsal **c** Antenna **d** Antennule **e** Maxillule **f** Maxilla **g** Mandible **h** Maxilliped. Scale bars: 300 µm (**a**); 100 µm (**b**); 50 µm (**c–h**).

*Body* cyclopiform, with oval cephalothorax and cylindrical urosome (Fig. 3a). Total body length ranging from 1.1 to 1.5 mm (mean = 1.3 mm, n = 4); width ranging from 580 to 600 µm (mean = 590 µm, n = 4). Prosome consists of cephalothorax (first pedigerous somite incompletely separated by an indistinct furrow) and three free pedigerous somites. Rostral area covered with hyaline setules (not figured). Second and third pedigerous somites with epimeral areas slightly angular. Fourth pedigerous somite smaller than preceding ones, its epimeral areas much less expanded.

*Urosome* s-shaped when alive, with the genital double-somite drawn forward under the metasome and the postgenital somites in line with the prosome (Fig. 3a); 5-segmented, comprising fifth pedigerous somite, genital double-somite, and three free abdominal somites (Fig. 3b). In dorsal view, only the postgenital somites are visible. Leg 5-bearing somite bell-shaped, slightly wider than long.

*Genital double-somite* (Fig. 3b) narrow, squarish (200 × 200 µm); its dorsal length (120 µm) much shorter than its ventral length (200 µm). Paired genital apertures bipartite, each comprising ventrolateral copulatory pore and dorsolateral gonopore (oviduct opening); lateral margins nearly parallel. Each genital area with two minute setae (Fig. 3b). Egg sac unknown. Width and length of three postgenital somites, 120 × 180, 85 × 130 and 105 × 120 μm from anterior to posterior.

*Caudal rami* (Fig. 3b) elongated, 180 × 45 µm, 4.0 times longer than wide. With six setae relatively short and naked. Outer lateral seta 52 µm, outermost terminal seta 41 µm, innermost terminal seta 47 µm. Two median terminal setae broadened, 58 µm (outer) and 52 µm (inner) in length. Dorsal seta 35 µm.

*Antennule* (Fig. 3d) 7-segmented, segments 67, 97, 41, 39, 35, 21 and 20 µm long respectively (measured along their posterior margin). Armature formula as follows: 1, 13, 6, 3, 4 and 1 aesthetasc, 3 and 1 aesthetasc and 7 (two of them joined at the base) and 1 aesthetasc. All setae relatively short and naked.

*Antenna* (Fig. 3c) 3-segmented; first segment 81µm long with small terminal hyaline seta; second segment 113 µm long with similar seta medially; third segment (formed by fusion of original segments 3 and 4 in *Lichomolgus*) 63 µm long with three hyaline setae medially (representing the usual three setae on penultimate segment in *Lichomolgus*) and two apical hyaline setae. Small recurved terminal claw 32 µm long. Length ratio of second to third segment (measured along inner margin) 2.1:1.

*Mandible* (Fig. 3g). Basal region with a rounded hyaline expansion and a distal row of small teeth on inner margin, and a fringe of setules on the outer margin. Terminal lash long, denticulated.

*Maxillule* (Fig. 3e) a single segment with a small seta and three hyaline prolongations (seemingly not articulated), one of them ornamented with setules.

*Maxilla* (Fig. 3f) 2-segmented; proximal segment unarmed; distal segment with a small seta medially, and two setiform processes apically, one barbed, the other with spinules.

*Maxilliped* (Fig. 3h) 3-segmented; first segment unarmed; second segment robust, with two naked inner setae; third segment claw-like denticulated distally, with two setae medially.

**Figure 4.**
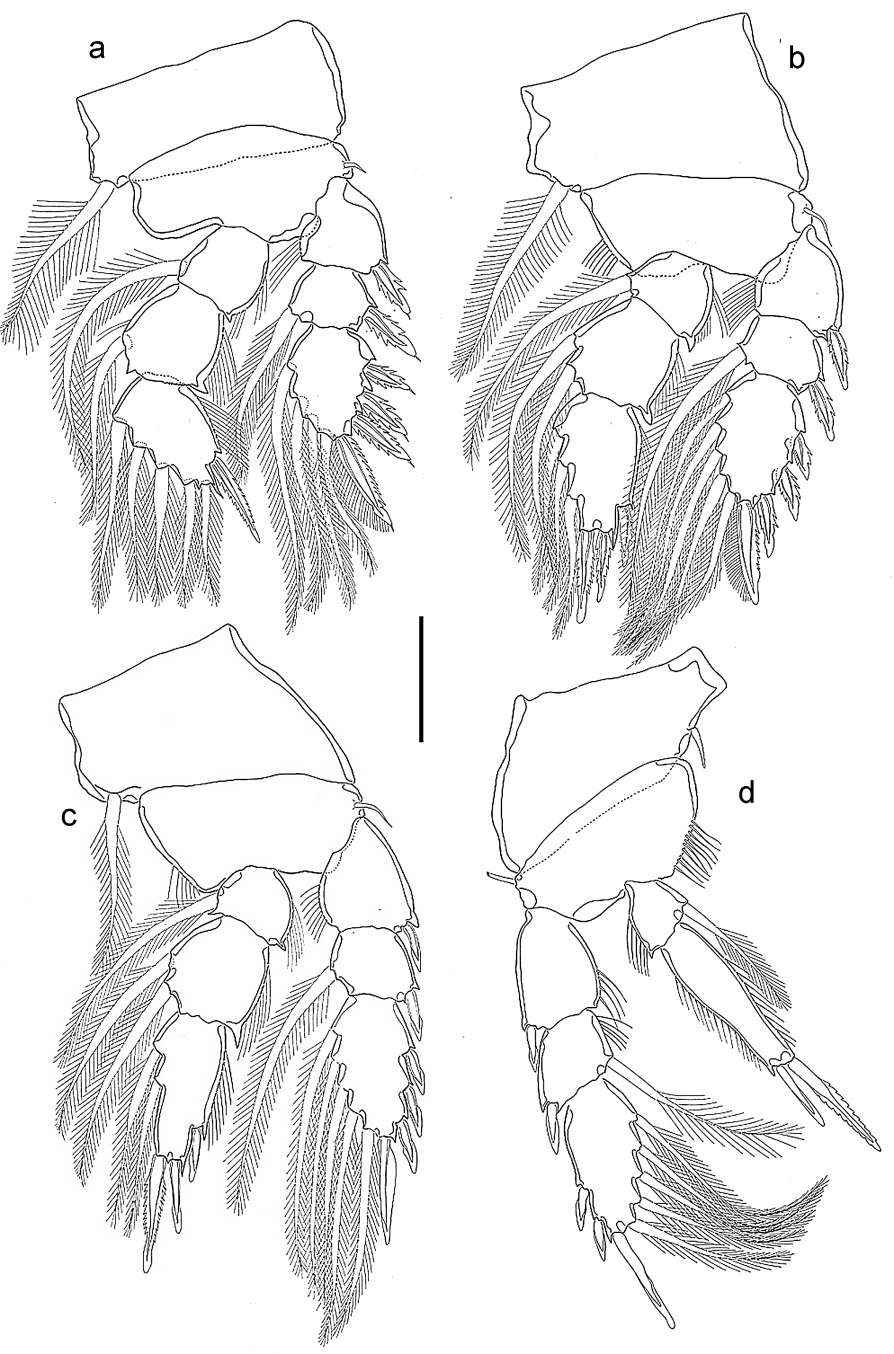
*Spaniomolgusglobus* sp. n., female. **a** Leg 1 **b** Leg 2 **c** Leg 3 **d** Leg 4 Scale bar: 50 µm.

*Legs 1–4* (Fig. 4a-d) with 3-segmented rami except for 2-segmented Sixth leg 4 endopod. Inner coxal seta long and plumose in legs 1–3, short and naked in Sixth leg 4. Outer basal seta short and naked in all legs. Endopod of Sixth leg 4 reaching beyond middle of third exopodal segment; with two terminal spines unequal in length, outer 32 µm long, inner 55 µm long, the latter spines with hyaline. Outer spines on Sixth leg 4 exopod with smooth lamellae. Armature formula as follows:

**Table:** 

	**Coxa**	**Basis**	**Exopod**	**Endopod**
Leg 1	0–1	1–0	I-0; I-1; III,I,4	0–1; 0–1; I,1,4
Leg 2	0–1	1–0	I-0; I-1; III,I,5	0–1; 0–2; I,II,3
Leg 3	0–1	1–0	I-0; I-1; III,I,5	0–1; 0–2; I,II,2
Leg 4	0–1	1–0	I-0; I-1; II,I,5	0–1; 0,II,0

*Fifth Sixth leg* (Fig. 3b) with protopod incorporated into somite; outer basal smooth seta minute. Free exopodal segment long, slender and recurved, 6.7 times as long as wide, bearing two apical setae unequal in length, innermost more than twice the length of outer one.

*Sixth leg* (Fig. 3b) represented by two very small articulated spines near attachment of eggs sacs.

Male unknown.

###### Spaniomolgus dentatus sp. n.

Taxon classificationAnimaliaCyclopoidaRhynchomolgidae

http://zoobank.org/4A6D3CC9-2492-4092-82D8-38F95675696A

Fig. 5

####### Type locality.

Saudi Arabian Red Sea, reef near Thuwal, 22°03'48.5"N, 38°45'51.2"E.

####### Material examined.

1 ♀ holotype (ZMMU Me-1213) and 1 ♀ paratype (ZMMU Me-1214) from *Stylophorapistillata* (morphotype *S.danae*) (KAUST SA2013-31) collected at 28 m depth.

####### Etymology.

The specific name from the Latin *dentatus*, refers to the denticulated margin of the maxillipedal claw.

####### Description.

Adult female.

**Figure 5.**
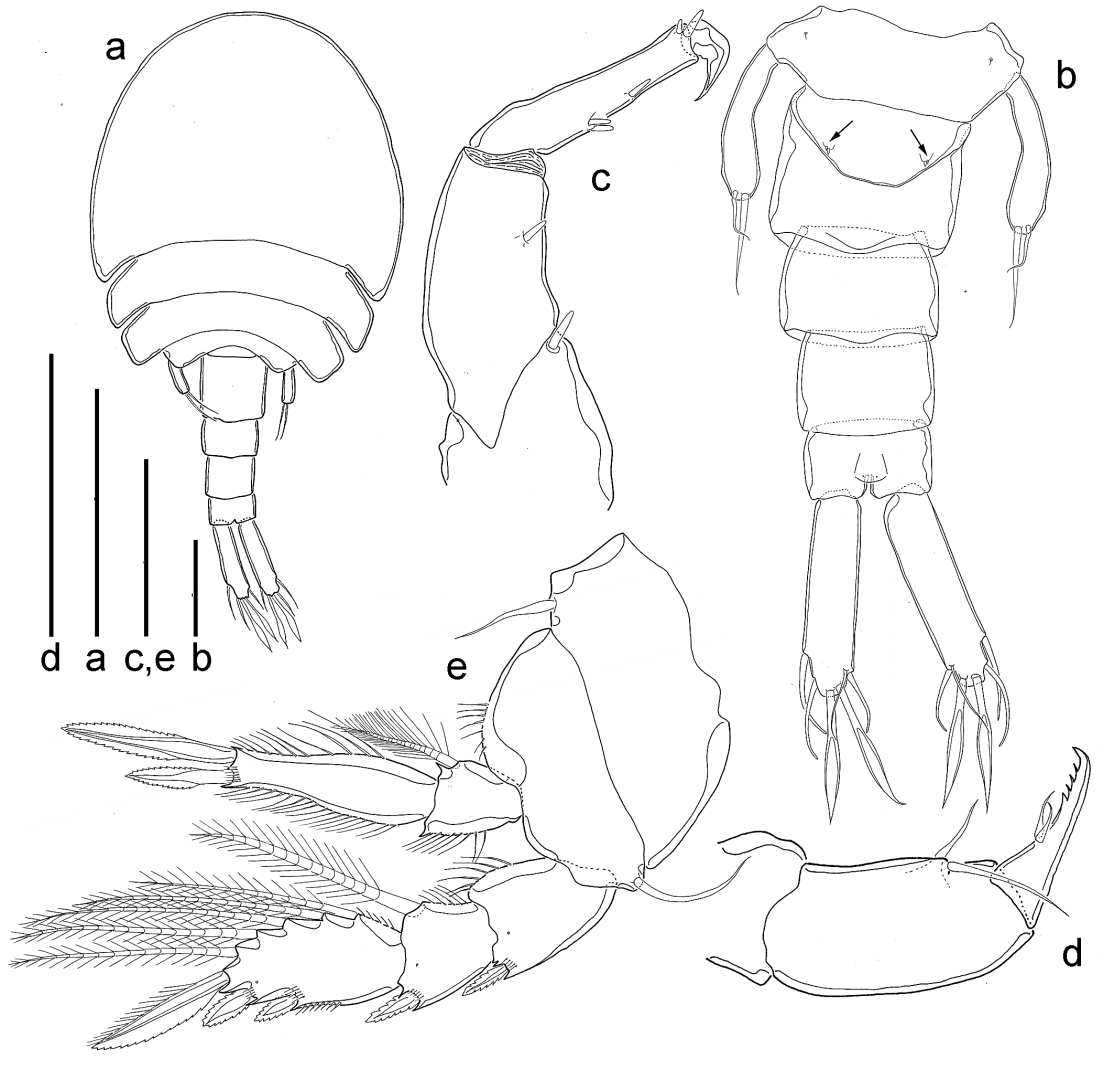
*Spaniomolgusdentatus* sp. n., female. **a** Habitus dorsal **b** Urosome dorsal (Leg 6 arrowed) **c** Antenna **d** Maxilliped **e** Leg 4. Scale bars: 300 µm (**a**); 100 µm (**b**); 50 µm (**c–e**).

*Body* cyclopiform, with oval cephalothorax and cylindrical urosome (Fig. 5a). Body length 750 µm and maximum width 390 µm. Prosome comprising cephalothorax and three free pedigerous somites. Second and third pedigerous somites with slightly rectangular epimeral areas. Fourth pedigerous somite smaller than preceding ones, its epimeral areas much less expanded.

**Figure 6.**
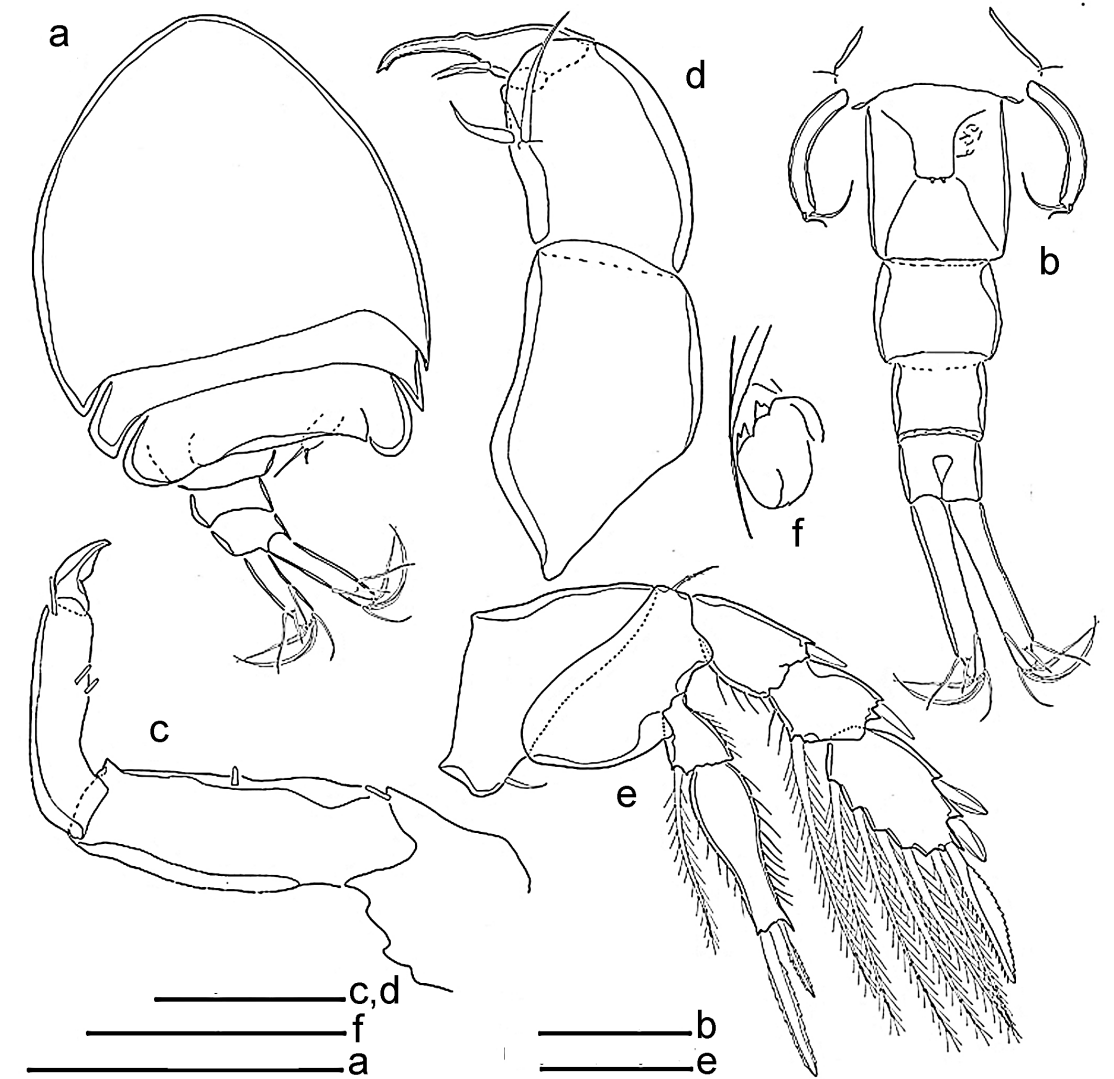
*Spaniomolgusmaculatus* sp. n., female. **a** Habitus dorsal **b** Urosome dorsal **c** Antenna **d** Maxilliped **e** Leg 4 **f** Genital area. Scale bars: 300 µm (**a**); 100 µm (**b**); 50 µm (**c–f**).

*Urosome* 5-segmented, comprising fifth pedigerous somite, genital double-somite and three free abdominal somites (Fig. 6b). Leg 5-bearing somite wider than long. Genital double-somite (Fig. 5b) slightly longer than wide (95 × 83 µm); lateral margins nearly parallel. Paired genital apertures bipartite, each comprising ventrolateral copulatory pore and dorsolateral gonopore (oviduct opening). Each genital area with two minute spiniform elements (Fig. 5b). Egg sac unknown. Three postgenital somites 55 × 83, 53 × 72 and 39 × 67 μm from anterior to posterior.

*Caudal rami* (Fig. 5b) elongated, 108 × 25 µm, 4.3 times as long as wide. With six setae; all setae relatively short and naked. Outer lateral seta 44 µm, outermost terminal seta 41 µm, innermost terminal seta 33 µm. Two median terminal setae broadened, 72 µm (outer) and 66 µm (inner) in length. Dorsal seta 39 µm.

*Antennule, mandible, maxillule, maxilla* and armature formula for legs 1–4 as for *Spaniomolgusglobus* sp. n.

*Antenna* (Fig. 5c) 3-segmented; first segment 53 µm long with small terminal hyaline seta; second segment 68 µm long with seta medially; third segment 60 µm long with three hyaline setae medially and two apical hyaline setae, small recurved terminal claw 24 µm long. Second and third segments measured along inner margin subequal in length.

*Maxilliped* (Fig. 5d) 3-segmented. First segment unarmed; second segment slightly elongated, with two naked inner setae; third segment claw-like, denticulate distally, with two setae medially.

*Leg 4* (Fig. 5e) with 3-segmented exopod and 2-segmented endopod. Inner coxal seta and outer basal seta naked. Endopod reaching beyond middle of third exopodal segment; second segment with two apical spines unequal in length, outer 30 µm long, inner 50 µm long, the latter spines with hyaline and weakly serrated margins. Outer spines of exopod with barbed lamellae.

*Fifth Sixth leg* (Fig. 5b) with protopod incorporated into somite; outer basal seta not observed. Free segment long, slender and recurved, 4.2 times as long as wide, bearing two apical setae unequal in length, inner most about twice as long as outer one.

*Sixth leg* (arrowed in Fig. 5b) represented by two very small articulated projections near attachment of eggs sacs.

Male unknown.

###### Spaniomolgus maculatus sp. n.

Taxon classificationAnimaliaCyclopoidaRhynchomolgidae

http://zoobank.org/3269010E-C96D-4F9B-8FBB-4189C01F6455

Fig. 6

####### Type locality.

Saudi Arabian Red Sea, reef near Thuwal, 22°19'09.26"N, 38°51'15.78"E.

####### Material examined.

1 ♀ holotype (ZMMU Me-1215) and 1 ♀ paratype (ZMMU Me-1216) from *Stylophorapistillata* (morphotype *S.subseriata*) (KAUST SA2013-25) collected at 10.4 m depth; 1 additional ♀ from *Stylophorapistillata* (morphotype *S.danae*) (KAUST SA2013-31) (22°03'48.5"N, 38°45'51.2"E) collected at 28 m depth.

####### Etymology.

The specific Latin epithet *maculatus* refers to the maculate body surface, light brown when alive.

####### Description.

Adult female.

*Body* cyclopiform; oval cephalothorax slightly pointed on top and cylindrical urosome (Fig. 6a). Mean body length 710 µm (with range of 700 – 720 µm) and mean maximum width 315 µm (with range of 270 – 360 µm), based on two specimens. Prosome comprising cephalothorax and three free pedigerous somites. Second pedigerous somite with epimeral area slightly angular and third pedigerous somite with epimeral area rounded. Fourth pedigerous somite smaller than preceding ones, almost invisible in dorsal view.

*Urosome* s-shaped when alive, with the genital double-somite drawn forward under the metasome and the postgenital somites retained in line with the prosome. Urosome 5-segmented, comprising fifth pedigerous somite, genital double-somite and three free abdominal somites (Fig. 6b). In dorsal view, only the postgenital somites visible. Leg 5-bearing somite slightly wider than long. Genital double-somite (Fig. 6b) narrow, slightly longer than wide (108 × 92 µm); lateral margins nearly parallel. Paired genital apertures bipartite, each comprising ventrolateral copulatory pore and dorsolateral gonopore (oviduct opening). Each genital area with two very small articulated projections (Fig. 6f). Egg sac unknown. Three postgenital somites 67 × 83, 50 × 63 and 42 × 54 μm from anterior to posterior.

*Caudal rami* (Fig. 6b) elongated, 125 × 21 µm, 5.0 times longer than wide. With six setae, all short and naked. Outer lateral seta 42 µm, outermost terminal seta 54 µm, inner lateral seta 33 µm, innermost terminal seta 37 µm, median terminal setae 71 µm in length. Dorsal seta 20 µm.

*Antennule, mandible, maxillule, maxilla* and armature formula for legs 1–4 as for *Spaniomolgusglobus* sp. n.

*Antenna* (Fig. 6c) 3-segmented; first segment 45 µm long with small hyaline apical seta; second segment 87 µm long with one hyaline seta medially; third segment 55 µm long with two hyaline setae medially, and one apical hyaline seta, with small recurved terminal claw 22 µm long. Length ratio of second to third segments (measured along inner margin) 1.7:1.

*Maxilliped* (Fig. 6d) 3-segmented; first segment unarmed; second segment robust, with two naked inner setae; third segment claw-like, with two setae medially equal in length; apex with pore.

*Leg 4* (Fig. 6e) with 3-segmented exopod and 2-segmented endopod. Inner coxal seta short and naked, outer basal seta short and plumose. Endopod reaching beyond middle of third exopodal segment; with two distal spines unequal in length, outer 30 µm long, inner 50 µm long, the latter spines with hyaline and weakly serrated margins. Outer spines of exopod with smooth lamellae.

*Fifth Sixth leg* (Fig. 6b) with protopod incorporated into somite; outer basal smooth seta short. Free segment long, slender and recurved, 7.6 times as long as wide, bearing two apical setae unequal in length, inner most about twice as long as outer one.

Male unknown.

###### Spaniomolgus acutus sp. n.

Taxon classificationAnimaliaCyclopoidaRhynchomolgidae

http://zoobank.org/10C25D5C-ED4B-4234-B6BA-F0B3988225B7

Fig. 7

####### Type locality.

Saudi Arabian Red Sea, reef near Thuwal, 22°19'9.26"N, 38°51'15.78"E.

Material examined. 1 ♀ holotype (ZMMU Me-1217) and 1 ♀ paratype (ZMMU Me-1218) from *Stylophorapistillata* (morphotype *S.subseriata*) (KAUST SA2013-25) collected at 10.4 m depth; 1 additional ♀ from *Stylophorapistillata* (morphotype *S.danae*) (KAUST SA2013-31) (22°03'48.5"N, 38°45'51.2"E) collected at 28 m depth.

####### Etymology.

The specific Latin epithet *acutus*, pointed, refers to the pointed epimeral areas of the second and third pedigerous somites.

####### Description.

Adult female.

**Figure 7.**
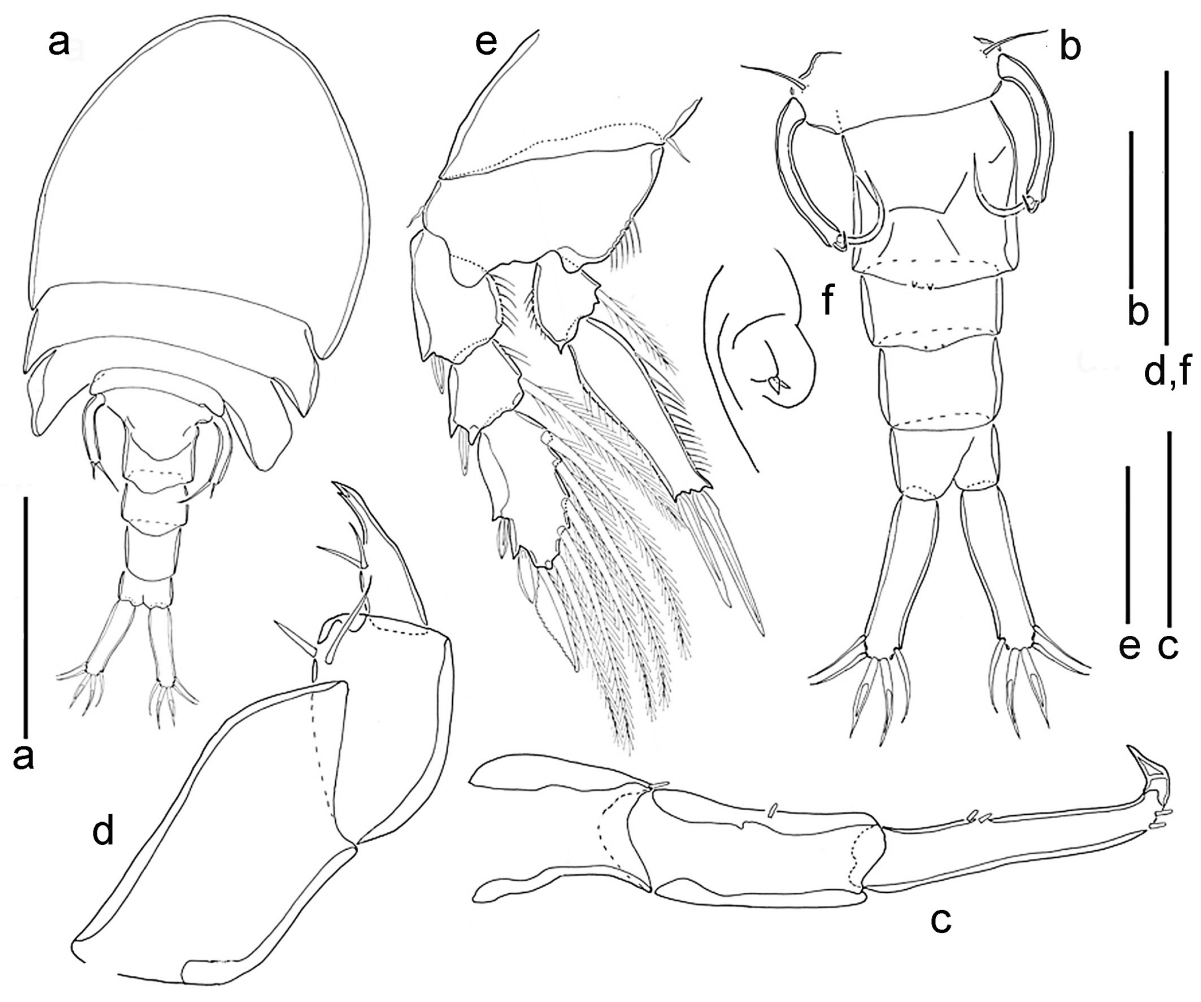
*Spaniomolgusacutus* sp. n., female. **a** Habitus dorsal **b** Urosome dorsal **c** Antenna **d** Maxilliped **e** Leg 4 **f** Genital area. Scale bars: 300 µm (**a**); 100 µm (**b**); 50 µm (**c–f**).

*Body* cyclopiform, with oval cephalothorax and cylindrical urosome (Fig. 7a). Mean body length 855 µm (with range of 850 – 860 µm) and mean maximum width 365 µm (with range of 320 – 410 µm), based on two specimens. Prosome comprising cephalothorax and three free pedigerous somites. Second and third pedigerous somites with epimeral areas pointed. Fourth pedigerous somite smaller than preceding ones, its epimeral areas much less expanded.

*Urosome* 5-segmented, comprising fifth pedigerous somite, genital double-somite and three free abdominal somites (Fig. 7b). Leg 5-bearing somite slightly wider than long. Genital double-somite (Fig. 7b) narrow, slightly longer than wide (107 × 100 µm); lateral margins nearly parallel. Paired genital apertures bipartite, each comprising ventrolateral copulatory pore and dorsolateral gonopore (oviduct opening). Each genital area with two minute spiniform elements (Fig. 7f). Egg sac unknown. Three postgenital somites 48 × 89, 52 × 78 and 40 × 70 μm from anterior to posterior.

*Caudal rami* (Fig. 7b) elongated, 111 × 30 µm, 3.7 times longer than wide. With five setae, all relatively short and naked. Outer lateral seta 44 µm, outermost terminal seta 41 µm, innermost terminal seta 48 µm. Two median terminal setae broadened, 52 µm (outer) and 59 µm (inner) in length. Dorsal seta not observed.

*Antennule, mandible, maxillule, maxilla* and armature formula for legs 1–4 as for *Spaniomolgusglobus* sp. n.

*Antenna* (Fig. c) 3-segmented; first segment 48µm long with small terminal hyaline seta; second segment 60 µm long, with similar seta medially; third segment 76 µm long, with two hyaline setae medially, and two apical hyaline setae, with small recurved terminal claw 20 µm long. Length ratio of second to third segments (measured along inner margin) 1:1.2.

*Maxilliped* (Fig. 7d) 3-segmented; first segment unarmed; second segment robust, with two naked inner setae; third claw-like segment with two setae medially, and a tooth subapically.

*Leg 4* (Fig. 7e) with 3-segmented exopod and 2-segmented endopod. Inner coxal seta and outer basal seta short and naked. Endopod reaching tip of third exopodal segment, with two apical spines unequal in length, outer 39 µm long, inner 52 µm long, the latter spines with hyaline and smooth margins. Outer spines on Sixth leg 4 exopod with smooth lamellae.

*Fifth Sixth leg* (Fig. 7b) with protopod incorporated into somite; outer basal seta smooth. Free segment long, slender and recurved, 9.3 times as long as wide, bearing two apical setae unequal in length, inner most 3.6 times the length of outer one.

*Sixth leg* (Fig. 7f) represented by two very small articulated projections near attachment of eggs sacs.

Male unknown.

###### Spaniomolgus stylophorus sp. n.

Taxon classificationAnimaliaCyclopoidaRhynchomolgidae

http://zoobank.org/56C93061-E2C5-47E5-8A3C-977D264B169E

Figs 8 , 9 b–d

####### Type locality.

Saudi Arabian Red Sea, reef near Thuwal, 22°12'04.30"N, 38°57'31.40"E.

####### Material examined.

1 ♀ holotype (ZMMU Me-1211) and 1 ♀ paratype (ZMMU Me-1212) from *Stylophorapistillata* (KAUST SA2013-12) collected at 1 m depth in the inner part of the reef; 1 additional ♀ from *Stylophorapistillata* (morphotype *S.danae*) (KAUST SA2013-31) collected at 28 m depth in the outer part of reef (22°20'23.45"N, 38°50'52.33"E).

####### Etymology.

The specific epithet *stylophorus* refers to the host name *Stylophora*.

####### Description.

Adult female.

**Figure 8.**
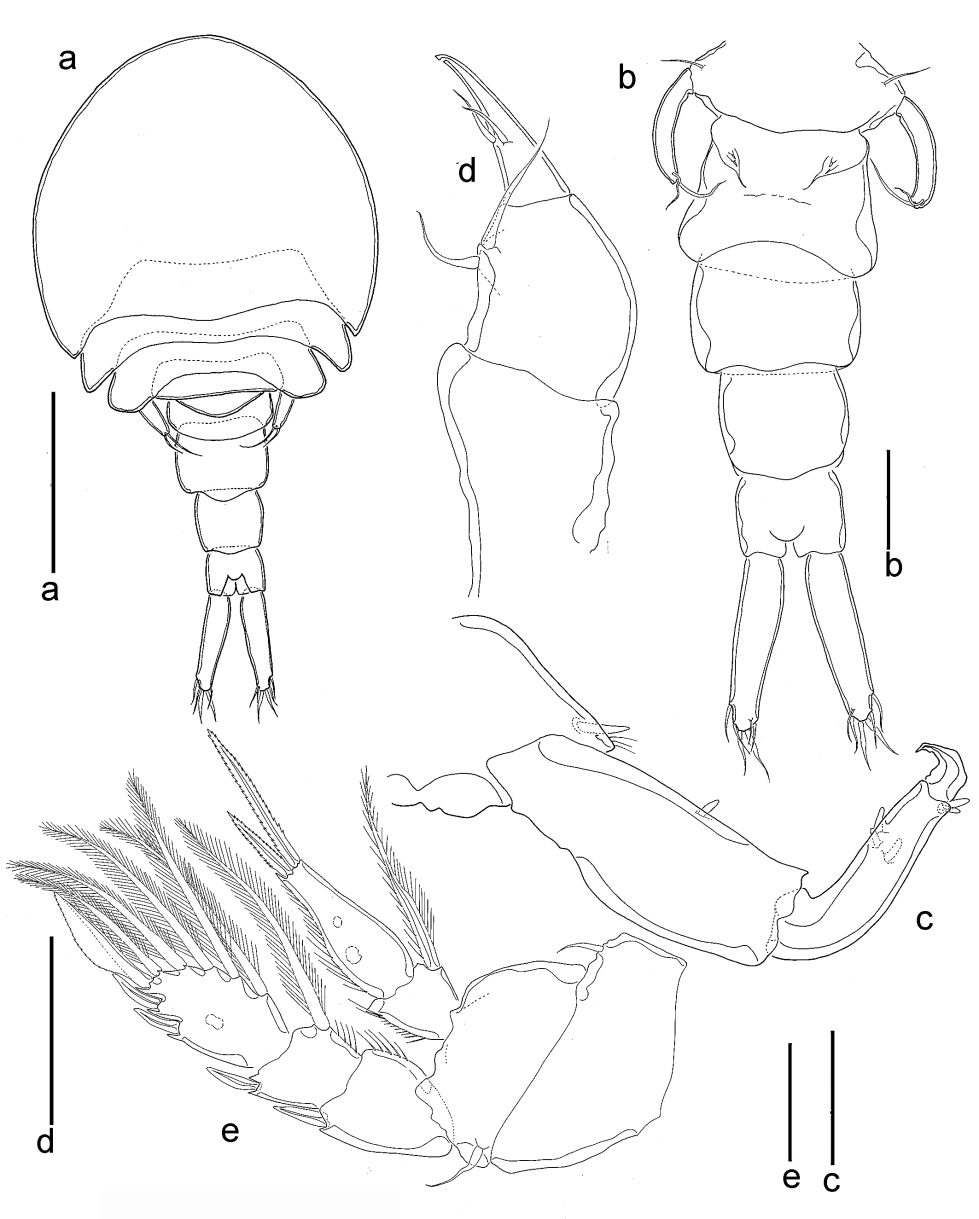
*Spaniomolgusstylophorus* sp. n., female. **a** Habitus dorsal **b** Urosome dorsal **c** Antenna **d** Maxilliped **e** Leg 4. Scale bars: 300 µm (**a**); 100 µm (**b**); 50 µm(**c–e**).

**Figure 9.**
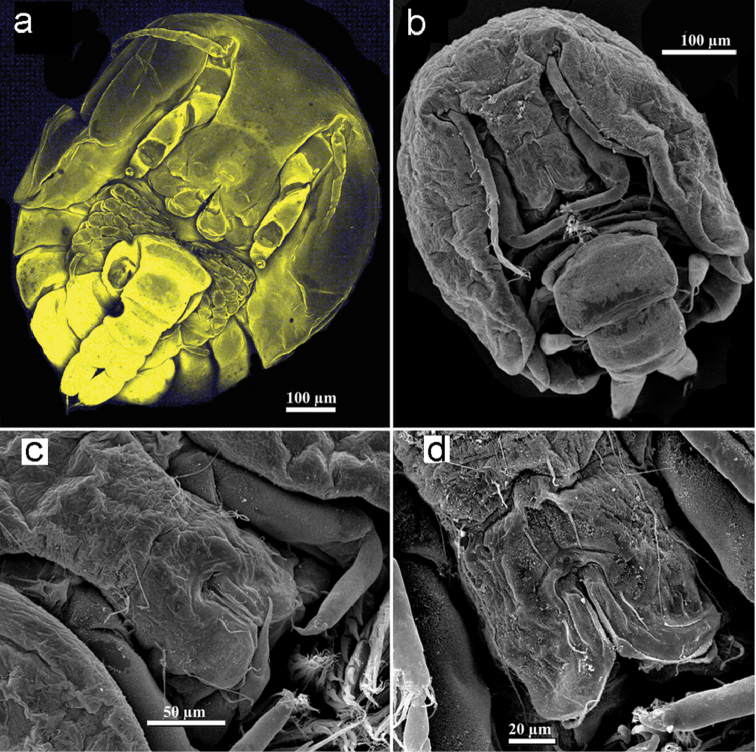
*Spaniomolgus*, females. **a***S.crassus* (Humes & Ho, 1968), confocal photo. *S.stylophorus* sp. n., SEM**b** Habitus ventral **c** Rostral area **d** Labrum.

*Body* cyclopiform, with oval cephalothorax and cylindrical urosome (Figs 8a, 9b). Mean body length 1.15 mm (with range of 1.1 – 1.2 mm) and mean maximum width 365 µm (with range of 320 – 410 µm), based on two specimens. Somite bearing Sixth leg 1 completely separated from cephalosome. Epimeral areas of metasomal somites slightly angular. Fourth pedigerous somite smaller than preceding ones, its epimeral areas not visible in dorsal view.

*Urosome* 5-segmented, comprising fifth pedigerous somite, genital double-somite and three free abdominal somites (Fig. 8b). In dorsal view, only the postgenital somites visible. Leg 5-bearing somite slightly wider than long. Genital double-somite (Fig. 8b) bell-shaped; 170 µm minimum width (anterior half), 220 µm maximum width (posterior half) and 155 µm long; shorter dorsally than ventrally. Paired genital apertures bipartite, each comprising ventrolateral copulatory pore and dorsolateral gonopore (oviduct opening). Each genital area with two minute spiniform setae (Fig. 8b). Egg sac unknown. Three postgenital somites 120 × 180, 120 × 130 and 94 × 110 μm from anterior to posterior.

*Caudal rami* (Fig. 8b) elongated, 200 × 45 µm, 4.4 times as long as wide. With six setae, all relatively short and naked. Outer lateral seta 40 µm, outermost terminal seta 40 µm, innermost terminal seta 30 µm. Two median terminal setae broadened, 50 µm (outer) and 60 µm (inner) in length. Dorsal seta 25 µm.

*Rostral area* with hyaline setules (Fig. 9c, d).

*Antennule, mandible, maxillule, maxilla* and armature formula for legs 1–4 as for *Spaniomolgusglobus* sp. n.

*Antenna* (Fig. 8c) 3-segmented; first segment 80µm long with small terminal hyaline seta; second segment 115 µm long with a seta medially; third segment 78 µm long with three hyaline setae medially, and two apical hyaline setae, with small recurved terminal claw 30 µm long. Length ratio of second to third segments (measured along inner margin) 1.5:1.

*Maxilliped* (Fig. 8d) 3-segmented; first segment unarmed; second segment robust, with two naked inner setae; third segment claw-like, with two setae medially equal in length; apex with pore.

*Leg 4* (Fig. 8e) with 3-segmented exopod and 2-segmented endopod. Inner coxal seta and outer basal seta short and naked. Endopod reaching beyond middle of third exopodal segment, with two apical spines unequal in length, outer 38 µm and inner 70 µm, the latter spines with hyaline and serrated margins. Outer spines of exopod with smooth lamellae.

*Leg 5* (Fig. 8b) with protopod incorporated into somite; outer basal seta naked. Free segment long, slender and recurved, 5.0 times as long as wide, bearing two apical setae unequal in length, inner most more than twice the length of outer one.

Male unknown.

###### Spaniomolgus crassus

Taxon classificationAnimaliaCyclopoidaRhynchomolgidae

(Humes & Ho, 1968)

Fig. 9a

####### Material examined.

2 ♀♀ found in tubular-shaped modification of corallites of *Stylophorapistillata* (morphotype *S.mordax*) (KAUST SA2013-72) collected on a reef near Al Lith at 2.5 m depth (20°08'02"N, 40°05'59"E).

## Discussion

### Taxonomy

Designation of the genus *Spaniomolgus* Humes & Stock, 1973 was based on three previously known species of *Lichomolgus* copepods associated with scleractinian corals: the type species *S.compositus*, *S.geminus*, and *S.crassus* from northern Madagascar (Humes and Frost 1964, Humes and Ho 1968). The finding of five new species and *S.crassus* in the Red Sea is the first record since 1968. Although *Spaniomolgus* is a rather homogenous genus, there are differences among its eight species.

The body has a broadened and thickened prosome in *S.crassus* and *S.globus*, but it is moderately widened, and the epimeral areas of the second and third pedigerous somites are slightly rectangular or angular in *S.stylophorus*, *S.geminus*, *S.compositus*, *S.dentatus*, *S.maculatus*, and *S.acutus*. Another key character to separate the species of *Spaniomolgus* is the body organization. For example, the first pedigerous somite is clearly set off from the cephalosome in *S.crassus* and *S.stylophorus*, incompletely separated from the cephalosome by an indistinct furrow in *S.geminus*, *S.compositus*, and *S.globus*, and completely fused to the cephalosome in *S.dentatus, S.maculatus*, and *S.acutus*.

The antennules are very similar in all eight species, with the only difference being the presence of an extra seta in the sixth segment in *S.globus*, *S.stylophorus*, *S.dentatus*, *S.maculatus*, and *S.acutus*.

The antenna of all species, except for *S.maculatus* and *S.acutus*, have the same armature formula (1,1,3+2+claw). *Spaniomolgusmaculatus* and *S.acutus* have a reduced armature of 1,1,2+1+claw and 1,1,2+2+claw, respectively. The length ratio of the second and the third segments of the antenna can be also used for species delimitation. For example, the length ratio of the two distal antennary segments is 1.1:1 in *S.crassus*, *S.geminus*, *S.compositus*, and *S.dentatus*, but 1.5:1 in *S.stylophorus*, 1.7:1 in *S.maculatus*, 2.1:1 in *S.globus* (2.1: 1), and 1:1.2 in *S.acutus*.

The maxillules of *S.globus*, *S.stylophorus*, *S.dentatus*, *S.maculatus*, and *S.acutus* are represented by a single segment bearing a small seta and three hyaline prolongations without evident articulation. However, according to Humes and Frost (1964) and Humes and Ho (1968), the maxillule shows four hyaline prolongations without articulation in *S.geminus*, *S.compositus*, and *S.crassus*. The condition of the maxillulary projections of the latter three species needs to be reassessed because the articulation of one of these elements was probably overlooked.

As for the maxilliped, small interspecific differences in the third claw-like segment were detected. The margin of the claw has three very small subterminal spinules in *S.geminus*, *S.compositus*, and *S.crassus*, but it is smooth and with an apical pore in *S.stylophorus* and *S.maculatus*. The distal half of the claw’s margin is denticulated in *S.globus* and *S.dentatus*; but with as single subapical tooth in *S.acutus*.

The armature of the legs is the same for the eight species; only the ornamentation of the fourth Sixth leg varies among the species. The exopodal spines have barbed lamellae in *S.geminus*, *S.compositus*, *S.dentatus*, *S.maculatus*, and *S.acutus*, but they are smooth in *S.crassus*, *S.globus*, and *S.stylophorus*. With respect to the terminal spines of the second endopodal segment, they are hyaline and smooth in *S.acutus* and *S.crassus*, but serrated in *S.stylophorus*, *S.dentatus*, *S.maculatus*, *S.compositus*, and *S.geminus*. In *S.globus* the outer terminal spine is serrated and the inner one is smooth.

The genital double-somite, generally rather narrow, can be present in three different shapes. In *S.crassus, S.compositus*, and *S.geminus* it is wider in its anterior third than in its posterior two-thirds; it is longer than wide with almost parallel margins in *S.dentatus, S.maculatus* and *S.acutus*, and completely square and bell-shaped in *S.globus* and *S.stylophorus* (wider in its posterior part).

The fifth Sixth leg in all species shows a long, slender and recurved segment of exopod with two apical setae. The length:width ratio of the free segment varies among the species, it is 10.5 times as long as wide in *S.geminus*, 9.3 times in *S.acutus*, 7.9 times in *S.compositus*, 7.6 times in *S.maculatus*, 6.7 times in *S.globus*, 6.3 times in *S.crassus*, 5.0 times in *S.stylophorus*, and 4.2 times in *S.dentatus*. Noteworthy, the outer basal seta of is minute in *S.globus* and has not been observed in *S.dentatus*.

The length:width ratio of the caudal rami, characteristically elongated in all the species, is also variable. The caudal rami are 9.1 times as long as wide in *S.geminus*, 5.0 times in *S.compositus* and *S.maculatus*, between 4.0 and 4.5 times in *S.globus*, *S.stylophorus* and *S.dentatus*, 3.7 times in *S.acutus*, and 2.8 times in *S.crassus*. The eight species present six terminal setae that are characteristically short and naked, except for *S.acutus* in which the dorsal seta has not been observed.

### Key to species of the genus *Spaniomolgus* Humes & Stock, 1973 (females)

**Table:** 

1	First pedigerous somite completely separated from cephalothorax	**2**
–	First pedigerous somite not completely separated from the cephalothorax	**3**
2	Prosome unusually broadened and thickened; caudal rami 2.8 times as long as wide; length ratio of second to third segments of the antenna 1.1:1; terminal claw of maxilliped with subterminal spinules	***S.crassus* (Humes & Ho, 1968)**
–	Prosome broad; caudal rami 4.4 times as long as wide; length ratio of second to third segments of the antenna 1.5:1; terminal claw of maxilliped with apical pore	***S.stylophorus* sp. n.**
3	First pedigerous somite incompletely separated from cephalosome by an indistinct furrow	**4**
–	Cephalosome fully incorporating first pedigerous somite	**6**
4	Caudal rami greatly elongated, 9.1 times as long as wide; outer exopodal spines of fourth Sixth leg with barbed lamellae; free segment of fifth Sixth leg 10.5 times as long as wide	***S.geminus* (Humes & Ho, 1968)**
–	Caudal rami 5.0 times as long as wide or less	**5**
5	Caudal rami 5.0 times as long as wide; length ratio of second to third segment of the antenna 1.1:1; outer exopodal spines of fourth Sixth leg with barbed lamellae; free segment of fifth Sixth leg 7.9 times as long as wide	***S.compositus* (Humes & Frost, 1964)**
–	Caudal rami 4.0 times as long as wide; length ratio of second to third segment of the antenna 2.1:1; outer exopodal spines of fourth Sixth leg with smooth lamellae; free segment of fifth Sixth leg 6.7 times as long as wide	***S.globus* sp. n.**
6	Outer exopodal spines of fourth Sixth leg with barbed lamellae; caudal rami 4.3 times as long as wide; length ratio of second to third segment of the antenna 1:1; free segment of fifth Sixth leg 4.2 times as long as wide	***S.dentatus* sp. n.**
–	Outer exopodal spines of fourth Sixth leg with smooth lamellae	**7**
7	Caudal rami 5.0 times as long as wide; length ratio of second to third segment of the antenna 1.7:1; free segment of fifth Sixth leg 7.6 times as long as wide; terminal claw of maxilliped with apical pore	***S.maculatus* sp. n.**
–	Caudal rami 3.7 times as long as wide; length ratio of second to third segment of the antenna 1:1.2; free segment of fifth Sixth leg 9.3 times as long as wide; terminal claw of maxilliped with a tooth subapically	***S.acutus* sp. n.**

## Supplementary Material

XML Treatment for Spaniomolgus

XML Treatment for Spaniomolgus globus

XML Treatment for Spaniomolgus dentatus

XML Treatment for Spaniomolgus maculatus

XML Treatment for Spaniomolgus acutus

XML Treatment for Spaniomolgus stylophorus

XML Treatment for Spaniomolgus crassus
